# Atmospheric Concentration of CO_2_ and PM_2.5_ at Salina, Stromboli, and Vulcano Islands (Italy): How Anthropogenic Sources, Ordinary Volcanic Activity and Unrests Affect Air Quality

**DOI:** 10.3390/ijerph19084833

**Published:** 2022-04-15

**Authors:** Paolo Madonia, Marianna Cangemi, Marcello Colajanni, Aldo Winkler

**Affiliations:** 1 Istituto Nazionale di Geofisica e Vulcanologia, Sezione di Roma 2, Via di Vigna Murata 605, 00143 Roma, Italy; aldo.winkler@ingv.it; 2 DiSTeM (Department of Earth and Marine Sciences), Università di Palermo, Via Archirafi 36, 90123 Palermo, Italy; mariannacangemi@gmail.com; 3 Petrochemical Engineering Consultant, 30033 Dolo, Italy; marcv@libero.it

**Keywords:** Aeolian Islands, gas hazard, magnetic properties, plant leaves, volcanic ash, volcanic unrest

## Abstract

Geogenic and anthropogenic sources of atmospheric particulate and CO_2_ can lead to threats to human health in volcanic areas. Although the volcanic CO_2_ hazard is a topic frequently debated in the related scientific literature, space and time distribution of PM_2.5_ are poorly known. The results of combined CO_2_/PM_2.5_ surveys, carried out at Salina, Stromboli, and Vulcano islands (Aeolian archipelago, Italy) in the years 2020–2021, and integrated with investigations on bioaccumulation of metallic particulate matter by the mean of data on the magnetic properties of oleander leaves, are presented in this work. The retrieved results indicate that no significant anthropogenic sources for both CO_2_ and PM_2.5_ are active in these islands, at the net of a minor contribution due to vehicular traffic. Conversely, increments in volcanic activity, as the unrest experienced by Vulcano island since the second half of 2021, pose serious threats to human health, due to the near-ground accumulation of CO_2_, and the presence of suspended micro-droplets of condensed hydrothermal vapor, fostering the diffusion of atmophile viruses, such as the COVID-19. Gas hazard conditions can be generated, not only by volcanic vents or fumarolic fields, but also by unconventional sources, such as the outgassing from shallow hydrothermal aquifers through drilled or hand-carved wells.

## 1. Introduction

As stated by the World Health Organization (WHO), “clear air is considered to be a basic requirement of human health and well-being” [1]. More than 2 million premature deaths each year are attributed to the adverse effects of outdoor/indoor urban air pollution, half of which affect people in developing countries [1].

Worldwide air quality guidelines (AQG) have been proposed by the WHO, for supporting actions to achieve air quality targeted to protect public health. However, each country formulates air quality standards based on national risk management and environmental policies, taking into account various aspects such as health risks, technological feasibility, and other economic considerations.

High levels of air pollution are usually recorded in the vicinity of specific sources, such as roads, power plants and other large stationary sources, including active volcanoes. Air pollutants can be released directly into the atmosphere, or as the result of chemical interactions involving precursor substances and including sources such as: volcanic eruptions, windblown dust, sea-salt spray, burning of fossil fuels, agriculture, waste treatment, industrial processes, etc. [2]. Recently, several key findings revealed that high air pollutant concentrations have health consequences (mostly mortality and morbidity), the most common of which are related to particulate matter (PM).

Moreover, PM can produce health effects both in short-term (24-h) and long-term (annual mean); WHO AQG values and interim targets (IT, intended as incremental steps in a progressive reduction of air pollution in areas where pollution is high) of PM are based on PM_2.5_. An annual average concentration of 5 µg m^−3^ was chosen as the long-term guideline values for PM_2.5_ [1]. In addition, four ITs are fixed at 35 (IT-1), 25 (IT-2), 15 (IT-3) and 10 (IT-4) µg m^−3^ for PM_2.5_ [1]. Similarly, short-term (24-h concentrations) AQG and IT for particulate matter were fixed by the WHO [1]. Short-term air quality guidelines are set at 15 µg m^−3^ for PM_2.5_, whereas corresponding IT values are 75, 50, 37.5 and 25 µg m^−3^ [1].

Most researches on PM exposure effects are focused on urban pollution, and few of these are related to volcanic activity [3,4,5]. A recent research, carried out at Kilauea volcano in the period 2007–2018, showed that during the 2018 eruption PM_2.5_ limits were exceeded for 34.7 and 46.3% of the time [5]. A study related to the 2010 Eyjafjallajökull eruption showed that high particulate matter concentrations were recorded during its explosive activity, exceeding the PM health limit by a factor more than 20 [3].

Freshly volcanogenic PM is highly heterogeneous in chemical composition, and can release great quantities of potentially harmful compounds, adsorbed onto ash surfaces as a consequence of the condensation/sublimation of volcanic acidic gases (i.e., sulfates and halides), metals and metalloids [6,7,8,9,10,11]. Moreover, salts formed by their reaction with the volcanic glass are readily soluble [12]. The particulate can be directly inhaled and/or ingested, causing respiratory pathologies and adverse effects to the digestive system, due to the release of soluble acidic compounds and metals, as in drinkable water, whose concentrations can exceed the acceptable daily intake (level of toxicity) established by the WHO [4].

Particle matter can show magnetic properties related to the presence of magnetite-like ferrimagnetic particles [13], associated with trace metals [14,15]. The magnetic fraction of PM may arise from exhaust processes related to industry, domestic heating, or vehicles, as well as from non-exhaust products from street surfaces and brake systems. Especially in urban areas, vehicular brakes represent the main source of PM associated with magnetite-like minerals (e.g., [16,17,18,19]).

Magnetic nanoparticles, which are common in urban airborne PM, were recently found even in human brain, where they can enter directly through the olfactory nerve. Thus, they are implicated in the production of damaging reactive oxygen species, which are causally linked to neurodegenerative diseases such as Alzheimer’s disease [20]. Exposure to up to ~22 billion magnetic nanoparticles/g of ventricular tissue appears to be directly associated with early and significant cardiac damage in children and young adults [21].

Rock magnetism methods have been widely applied to plant leaves and lichens for biomonitoring air pollution, being efficient PM receptors [22,23,24]. Magnetic properties detected on tree leaves and lichens mostly depend on the concentration and the grain size of magnetite-like minerals accumulated on the samples, with the magnetic susceptibility being the fastest, most sensitive and practical parameter [25], recently tested also for *in situ* detection of the time dependent variations of the concentration of magnetic particles [26].

Other air pollutants are in gaseous phase and, among these, carbon dioxide is one of the most harmful for humans. The adverse effects due to the inhalation CO_2_ depend on both concentration and exposure time: from headache (3% CO_2_ for 1 h), increased respiratory and heart rates, dizziness, muscle twitching, confusion, unconsciousness, coma (concentrations > 15% for 1 min), and finally death (concentrations exceeding 50%) [27]. Carbon dioxide is asphyxiant and odorless, and it can accumulate in depressed areas, especially under wind calm; for these reasons it is extremely insidious, deserving the appellation of “silent killer”.

Carbon dioxide is one of the main gases released to the atmosphere by volcanic activity, directly in the volcanic plume or through the soil, concentrated in fumaroles or by diffuse degassing [28,29]. The two most lethal events associated with volcanic CO_2_ emissions were recorded in 1979 in Indonesia, and in 1986 in Cameroun. The first one occurred on 20 February 1979 in Dieng Plateau (Indonesia), where 149 people died and other 1000 were injured because they were enveloped in a gas cloud generated by a phreatic eruption. The second one, the most lethal ever occurred worldwide, happened on 21 August 1986 at Lake Nyos (Cameroun), where a nighty CO_2_ outburst killed 1746 people, injuring other 845 [27] (and references therein).

Particularly high CO_2_ hazard levels characterize densely inhabited active volcanoes, with urbanized areas located in the close proximity of the emission points.

At Vulcano island, during the volcanic crisis of 1988, two children died of asphyxiation [29,30,31]; again, in April 2015, a 9-year-old French child remained seriously asphyxiated whilst approaching a shallow undersea vent close the shoreline of the Baia di Levante (East Bay) area [32].

**Figure 1:**
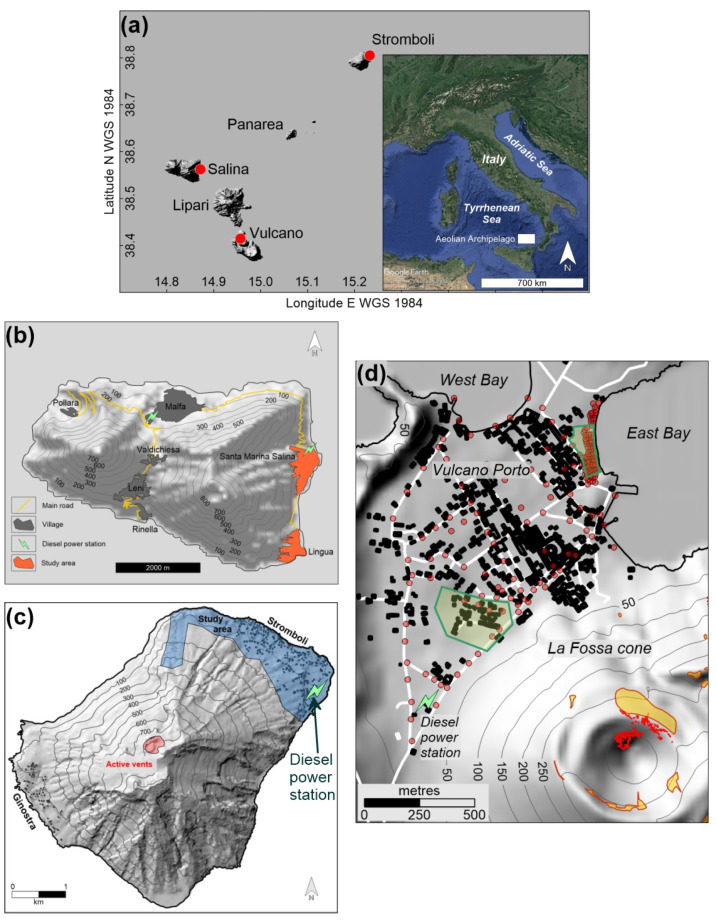
(**a**) Geographical setting of Salina, Stromboli and Vulcano islands; (**b**) Map of Salina island with location of the studied area; (**c**) Map of Stromboli island with location of the studied area; (**d**) Map of the Vulcano Porto area and La Fossa cone, at Vulcano island, with location of points of measure (red circles), anomalous soil CO_2_ degassing areas [36] (green polygons), main high (T > 100 °C) and low (T < 100 °C) temperature fumarolic fields (red and orange polygons, respectively).

The most recent, and hugest, gas hazard event ever recorded at Vulcano island is still ongoing: a massive CO_2_ release from soil and fumaroles, during a volcanic unrest started between July and September 2021, led first to the permanent evacuation of a restricted area on 19 October. Afterwards, due to the permanency of high CO_2_ emissions, an ordinance by the mayor of Lipari, issued on 20 November 2021, prohibited to stay overnight (from 11 p.m. to 6 a.m.) at the ground floor of all the buildings in the Vulcano Porto area, overwhelmed south-eastward by the active volcanic cone of La Fossa (Figure 1).

The 2021 CO_2_ degassing crisis at Vulcano island has occurred during the COVID-19 pandemic, started in Italy in February 2020, and contrasted by a prolonged forced confinement that, during the periods with major restrictions to the normal working and social activities, determined a reduction in the release of anthropogenic atmospheric pollutants worldwide [33,34].

This particular condition prompted, at the end of summer 2020, a study aimed to collect the first measures of PM_2.5_ and outdoor air CO_2_ concentrations in some inhabited areas of the volcanic Aeolian islands, focused on the spatial discrimination between their anthropogenic and geogenic sources. Several field surveys were carried out in the islands of Salina, where the last eruption took place 15 ka ago [35], Stromboli, with a permanently erupting volcano, and Vulcano, which has been characterized by a continuous fumarolic activity since its last eruption dated 1888–1890 (Figure 1), periodically experiencing degassing crises. Spot spatial measurements were integrated with investigations on magnetic properties of plant (oleander) leaves, which were tested for their ability of bioaccumulating atmospheric particulate, give information on time continuity of the observed phenomena.

## 2. Materials and Methods

Surveys were carried out during days characterized by stable atmospheric conditions, at the net of the normal circadian variations, and low wind field (absence of strong gusts and average wind speed < 5 m s^−1^). Meteorological data are from the closest available station representative of the topographic conditions of the surveyed areas: the INGV station of Stromboli, based on a Davis Instrument weather station and installed in the port area.

A handheld Temtop (Shangai, China) M2000 instrument was used for real-time measures of PM_2.5_ and CO_2_, which were taken at 1.5 m from the soil, and with variable durations in the order of tens of seconds until displayed concentrations were stable. The device is equipped with a laser PM sensor and a Non-Dispersive InfraRed (NDIR) carbon dioxide sensor. Measurement ranges are 0–999 µg m^−3^ for PM_2.5_, and 0–5000 ppm for CO_2_, 0–50 °C for temperature and 0–90% for Rh, with resolutions of 0.1 µg m^−3^ for PM_2.5_ and 1 ppm for CO_2_.

Measures were positioned using a Trimble (Sunnyvale, CA, USA) Juno SB, single frequency, handled GPS. Contour maps were draft applying the kriging algorithm implemented in Golden Software (Golden, CO, USA) Surfer, release 16. Thermal photos were taken using a FLIR (Willsonville, OR, USA) thermal camera.

Magnetic properties of leaves were determined collecting specimens from Oleander trees, which are commonly found in all the investigated areas. Dried leaf samples were placed into standard 8 cm^3^ palaeomagnetic plastic cubes for magnetic susceptibility analyses or fragmented inside pharmaceutical gel caps #4 for measuring the hysteresis loops.

Mass magnetic susceptibility (χ) was calculated, for individual samples, as the average value after 5 consecutive measurements by means of a KLY5 Agico meter and dividing the obtained values for the net weight of the samples. The coercive force (Bc), the saturation remanent magnetization by mass (Mrs, or SIRM) and the saturation magnetization by mass (Ms) were determined on selected samples with a vibrating sample magnetometer (Lakeshore 8604) at a maximum field of 1.0 T; concentration dependent hysteresis parameters were calculated subtracting the high field paramagnetic linear trend before dividing the magnetic moments for the net weight of the samples. The coercivity of remanence (Bcr) values were interpolated from backfield remagnetization curves up to −1 T, after saturating at 1 T field.

The domain state and magnetic grain-size of the samples were compared to theoretical magnetite according to the hysteresis ratios Mrs/Ms vs. Bcr/Bc in the “Day plot” [37,38,39], which were used to distinguish between single domain (SD), multidomain (MD) and pseudo-single domain (PSD) behaviors.

Simulations of the mixing between groundwater and CO_2_ were performed using the open-source chemical process simulator DWSIM 6.7.1 [40]. Dates and meteorological conditions during surveys are reported as daily averages in Table 1.

## 3. Study Area Setting

Salina, Vulcano and Stromboli islands belong to the Aeolian Archipelago in the Southern Tyrrhenian Sea (Figure 1a), located between Sicily and Calabria (Italy).

**Figure 2:**
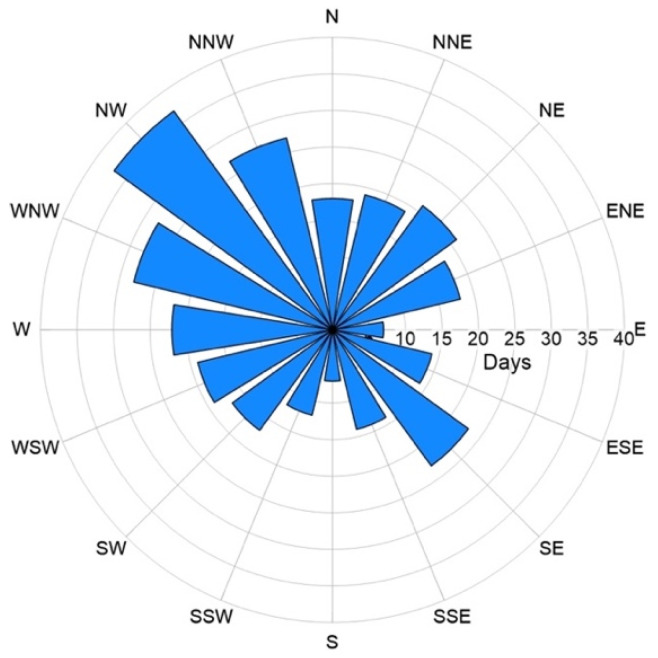
Wind rose diagram showing the prevalent wind direction in the eastern sector of the southern Tyrrhenian Sea [41].

The climate is typical of the Mediterranean, with warm, dry late springs-summers and mild, wet autumn-winters. Prevailing winds blow from the NW quadrant and, secondarily, from SE, with 62 days per year characterized by wind calm conditions (wind speed < 1 knot) [42] (Figure 2).

### 3.1. Salina

Salina (Figure 1b), with a maximum elevation of 962 m a.s.l., is the highest island of the archipelago, and as the other ones it is the emerged part of a larger stratovolcano rising from the sea bottom. Its total stable population is about 2300 people, living in the villages of Lingua, Santa Marina Salina (the most populated, with 850 inhabitants, and the main harbor of the island) Malfa, Valdichiesa, Leni, Rinella and Pollara.

The volcanological evolution of Salina comprises six Eruptive Epochs (EE), between 244 and 15.6 ka [35]. Products of the last EE are found in the Pollara crater (NW termination of the island), where rhyolitic pumiceous successions (30 and 15 ka) outcrop. Presently, there is a residual, mild hydrothermal activity: a shallow, low-enthalpy hydrothermal aquifer and some undersea degassing vents around the island.

Salina is the only island of the Aeolian Archipelago with an economy not based on tourism, but on the cultivation and transformation of high-quality land products, mainly capers and grapes, from which a famous sweet wine (Malvasia) is produced. There is vehicular traffic and electricity is produced by two thermal power stations, burning diesel fuel, located at Malfa and Santa Marina Salina, respectively (Figure 1b).

### 3.2. Stromboli

Stromboli (Figure 1c), one of the most active volcanoes of the world, rises up to 924 m a.s.l. It is permanently inhabited by 550 people, mostly concentrated in the north-eastern village of Stromboli. The other settlement, Ginostra, located on the southwestern side is populated by few tens of persons. However, during the summer touristic season, its population grows up to several thousand persons per day.

The rock composition varies among basaltic andesites, shoshonites, and latites-trachytes [43,44,45,46,47]. It is an open conduit, permanently active volcano, characterized by a peculiar (strombolian) continuous explosive activity, from vents located on the summit crater terrace (Figure 1c). The normal strombolian activity is characterized by passive magma degassing alternating with short-lasting (up to few tens of seconds) 100- to 200-m high scoria-rich jets, caused by variable energy explosions every 10–20 min [48,49,50]. This activity is occasionally interrupted by explosive events of higher intensity [51,52].

The vehicular traffic is scarce, limited to motorcycles and small three-wheeler vans (due to the very narrow streets), many of which powered by electric engines. There is a thermal power station, supplied by diesel fuel, located close to the sea in the Stromboli village (Figure 1c).

### 3.3. Vulcano

Vulcano (Figure 1d), the southernmost island of the archipelago, is permanently inhabited by 450 people but, due to its huge hospitality capacity (the highest among the Aeolian Islands), this number increase more than tenfold during the touristic summer season, and, consequently, the related vehicular traffic.

From the volcanological point of view, Vulcano has been built during six main stages of activity, producing high-K calc-alkaline (HKCA), shoshonitic (SHO), and leucite tephrite or potassic (KS) rocks, widely varying in their degree of evolution from basaltic to rhyolitic [53,54,55]. The morphological structure is characterized by two calderas: the “Piano Caldera”, constituting the southeastern sector of the island, and “La Fossa”, in the north-western sector. The most recent activity was produced by La Fossa cone (Figure 1d, 391 m a.s.l.), with a last eruption dated 1888–1890 A.D.

Since the last eruption, volcanic activity has been mainly solphataric, with a main high (T > 100 °C) fumarolic field located in the inner northern flank of the cone, whereas a low (T < 100 °C) temperature ring of fumaroles borders the crater rim (Figure 1d). Another exhaling area is located in the isthmus connecting the main body of the island with the little cone of Vulcanello; here, degassing vents are located both onshore and in the near offshore and due, to the high touristic frequentation of the area, were responsible in the past years of health issues and fatalities, as described in the introduction. Moreover, significant CO_2_ emissions are also due to intense diffuse soil degassing from other delimited areas inside the Vulcano Porto village [36], at whose south termination a diesel power station is also located (Figure 1d).

## 4. Results

### 4.1. PM_2.5_ Concentrations

**Figure 3:**
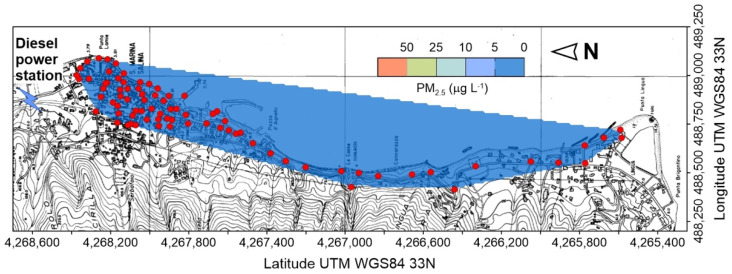
Concentration of PM_2.5_ at Santa Marina di Salina on 4 January 2021; red circles are points of measure.

**Figure 4:**
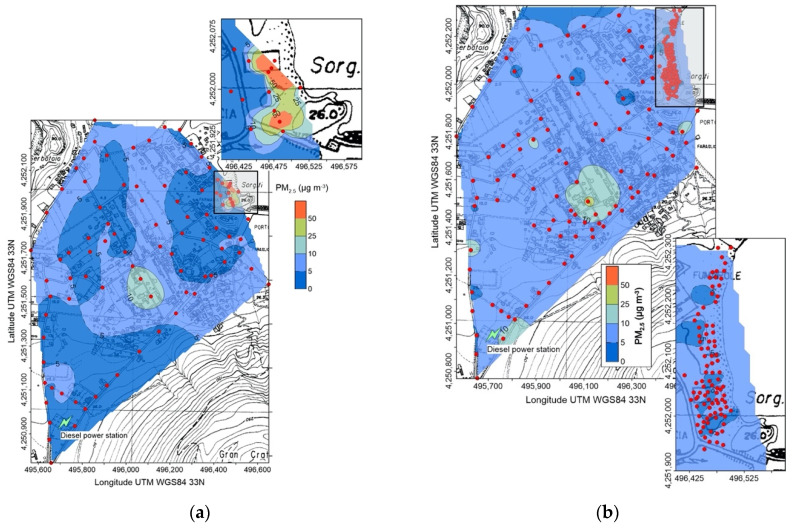
Concentration of PM_2.5_ in the Vulcano Porto area on 6 January 2021 (**a**) and 13 November 2021; (**b**) red circles are points of measure. The position of the power station is also shown. In the inset, detail of the East Bay area (grey rectangle in the main map).

**Figure 5:**
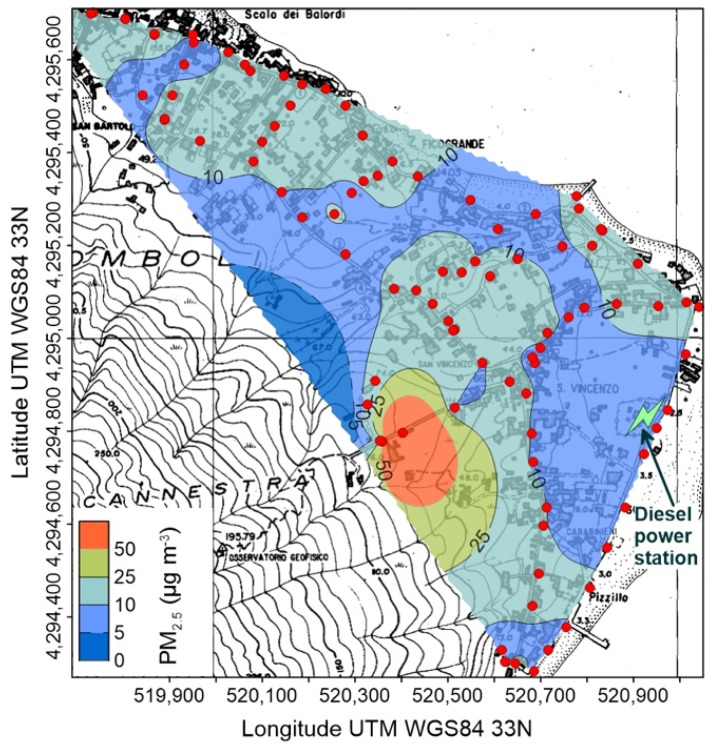
Concentration of PM_2.5_ at Stromboli on 23 September 2020; red circles are points of measure. The position of the power station is also shown.

**Figure 6:**
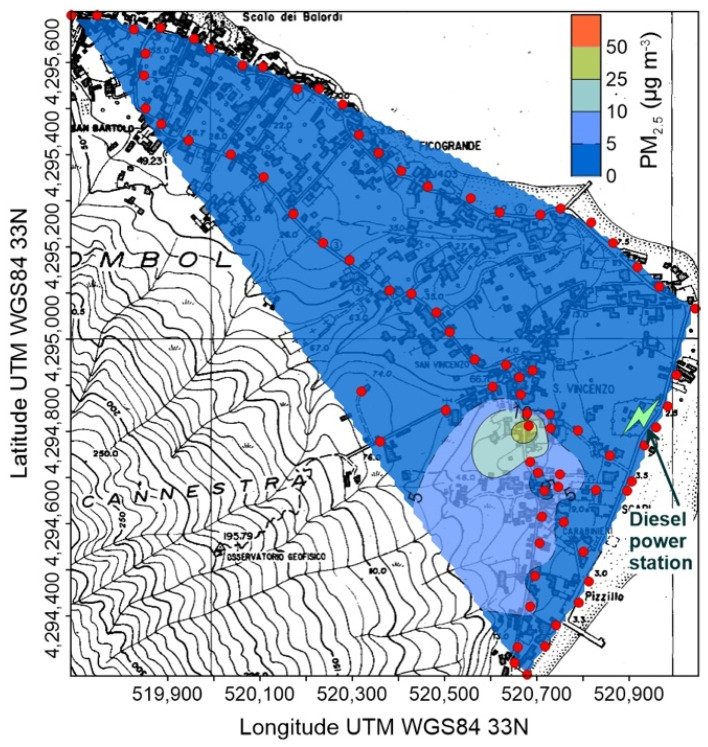
Concentration of PM_2.5_ at Stromboli on 5 January 2021; red circles are points of measure. The position of the power station is also shown.

**Figure 7:**
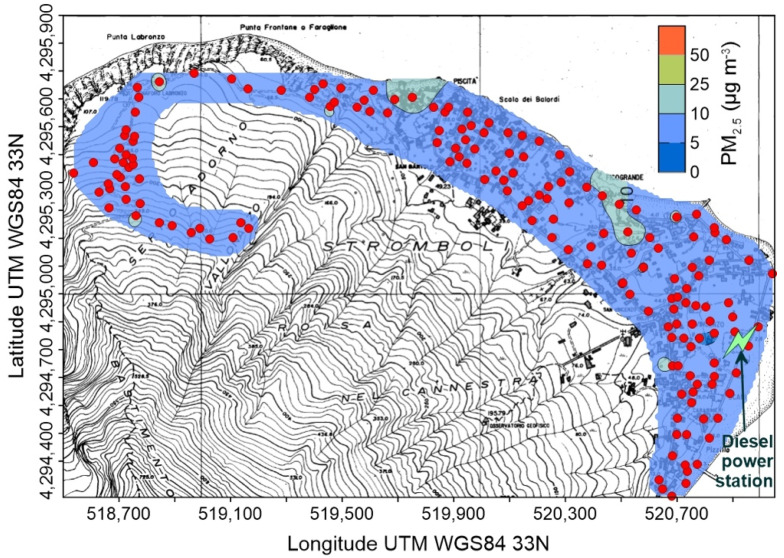
Concentration of PM_2.5_ at Stromboli on 2 April 2021; red circles are points of measure. The position of the power station is also shown.

**Figure 8:**
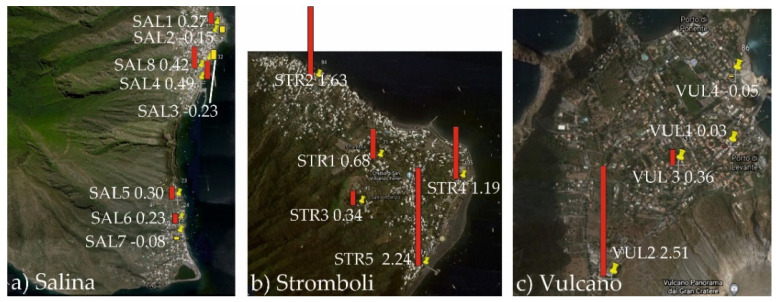
Magnetic Susceptibility values for leaves (χ, 10^−8^ m^3^ kg^−1^), represented as bars placed near the sampling sites (red, positive values; yellow, negative values), for Salina (**a**), Stromboli (**b**) and Vulcano (**c**) islands.

Several survey campaigns were carried out in the studied areas between September 2020 and November 2021: one at Salina (January 2021), three at Stromboli (September 2020, January and April 2021), and two at Vulcano (January and November 2021). Minimum, average and maximum concentrations of PM_2.5_ are reported in Table 2 (full data available as Supplementary Materials) and Figure 3, Figure 4, Figure 5, Figure 6, Figure 7 and Figure 8. Among the different islands, Salina (Figure 3) is the site showing the lowest concentrations and the lowest variability, with minimum, maximum, average and standard deviation at 0.3, 5.1, 1.66 and 1.02 µg m^−3^, respectively.

On the contrary, in the same period (January 2021), the highest average (13.6 µg m^−3^) and maximum (656 µg m^−3^) concentrations were recorded at Vulcano island, and in particular in the East Bay area, where a diffuse hydrothermal degassing is present, and along the road, oriented NW-SE, crossing the inhabited area of Vulcano Porto (Figure 4a); out of these areas, PM_2.5_ values were almost constant and lower than 10 µg m^−3^.

The replicated survey, on November 2021 (Figure 4b), confirmed the presence of higher concentrations along the road, the disappearance of the anomalous area of East Bay, but the appearance of PM_2.5_ values higher than 10 µg m^−3^ close to the diesel power station located at the SW corner of the inhabited area. Other single, scattered points with concentrations higher than the average are also present.

A huge variability of PM_2.5_ characterizes Stromboli. The highest values were recorded on September 2020 (Figure 5), with wide areas showing concentrations higher than 5 µg m^−3^, and reaching maxima higher than 50 µg m^−3^ in the central, uphill zone of the village; it is worth noting that these maxima were recorded shortly after a volcanic explosion from the summit craters.

The second survey, on January 2021 (Figure 6), depicted a completely different situation, with low (<5 µg m^−3^) PM_2.5_ concentrations and few higher values, concentrated in the southern, uphill corner of the inhabited area.

The third survey (April 2021, Figure 7) identified constant, slightly higher (between 5 and 10 µg m^−3^) concentrations, with the presence of two small zones with values >10 µg m^−3^, located along the perimetral coastal road of the village.

### 4.2. Magnetic Susceptibility and Hysteresis Properties

Magnetic susceptibility values are summarized in Table 3 and illustrated in Figure 8. They range, for the whole dataset, from −0.23 to 2.51 × 10^−8^ m^3^ kg^−1^, indicating, a low concentration of ferrimagnetic minerals. In particular, χ average values were 0.16 ± 0.10, 1.22 ± 0.34 and 0.72 ± 0.61 for Salina, Stromboli and Vulcano islands, respectively.

Only 4 sites recorded χ > 1 × 10^−8^ m^3^/kg values, indicative of the more relevant presence of magnetic particles. At Stromboli Island, these values were reached in a narrow street with low traffic intensity, in a perimeter coastal road near the thermoelectric power station and, above all, at the Scari pier, where the most of vehicular traffic concentrates.

The highest χ value (2.51 × 10^−8^ m^3^ kg^−1^) was recorded at Vulcano island, near the thermoelectric power plant.

**Figure 9:**
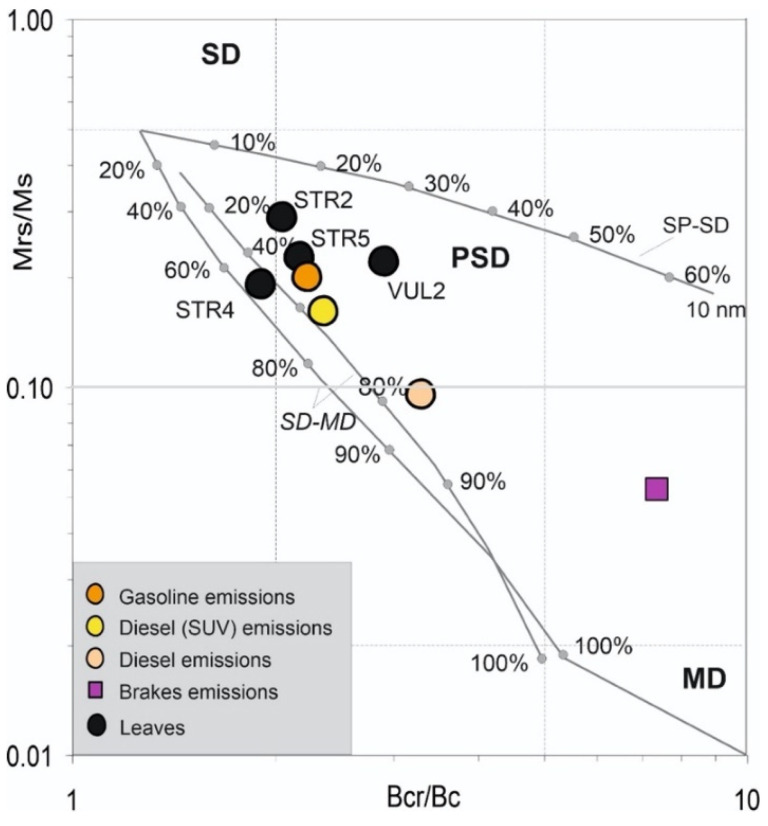
Bilogarithmic “Day Plot” for the leaves (black circles) sampled in Stromboli and Vulcano Islands, selected according to their magnetic susceptibility values. The orange, yellow and pink circles represent the average values for different kind of exhaust vehicular emissions (calculated from [55]). In magenta square, the non-exhaust brake emissions, averaged from [56].

Due to the low values of magnetic susceptibility, indicating prevailing paramagnetic behavior for most of the samples, the hysteresis properties were determined only for the samples with χ > 1 × 10^−8^ m^3^/kg, which are STR2, STR4, STR5 and VUL2, as reported in Table 4. Hysteresis loops easily saturated, highlighting prevailing ferrimagnetic behaviour, with coercivities in range with magnetite-like minerals. In the “Day plot” (Figure 9), the points fall in the central-upper area of the diagram, near fuel emissions and far from brakes, comparing the leaves to the exhaust and non exhaust vehicular emissions averaged from Sagnotti et al. [56].

### 4.3. CO_2_ Concentrations

**Figure 10:**
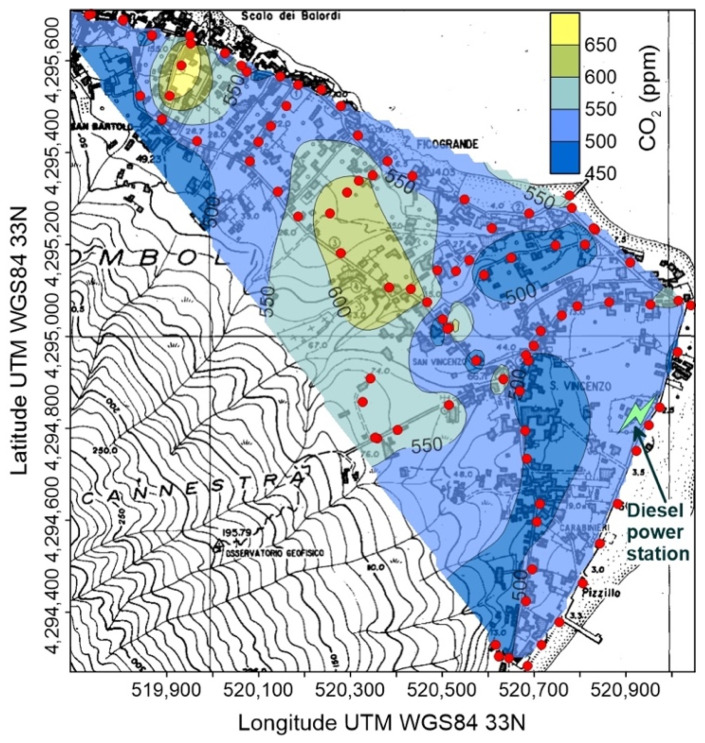
Concentration of atmospheric CO_2_ at Stromboli on 23 September 2020; red circles are points of measure. The position of the power station is also shown.

**Figure 11:**
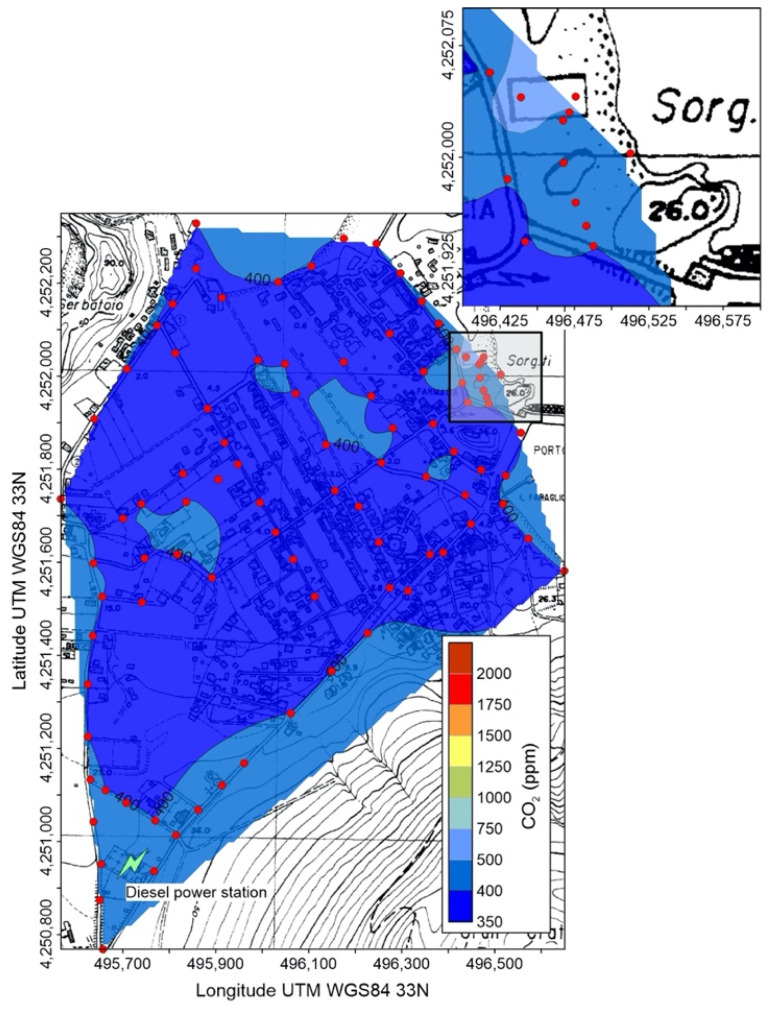
Concentration of atmospheric CO_2_ in the Vulcano Porto area on 6 January 2021; red circles are points of measure. The position of the power station is also shown. In the inset, detail of the East Beach area (grey rectangle in the main map).

**Figure 12:**
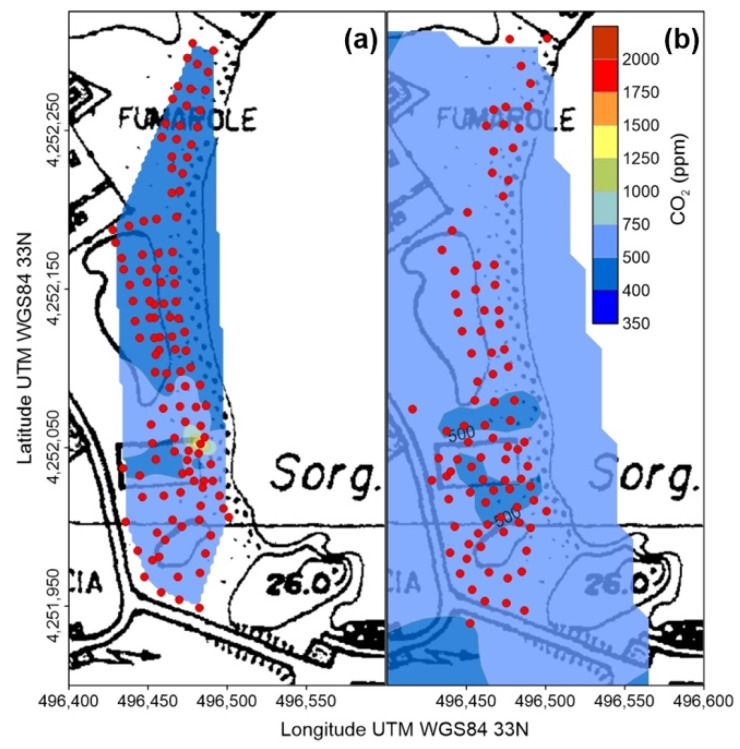
(**a**) Concentration of atmospheric CO_2_ in the East Bay of Vulcano Porto area on 13 October 2021; (**b**) Concentration of atmospheric CO_2_ in the East Bay of Vulcano Porto area on 13 November 2021.

**Figure 13:**
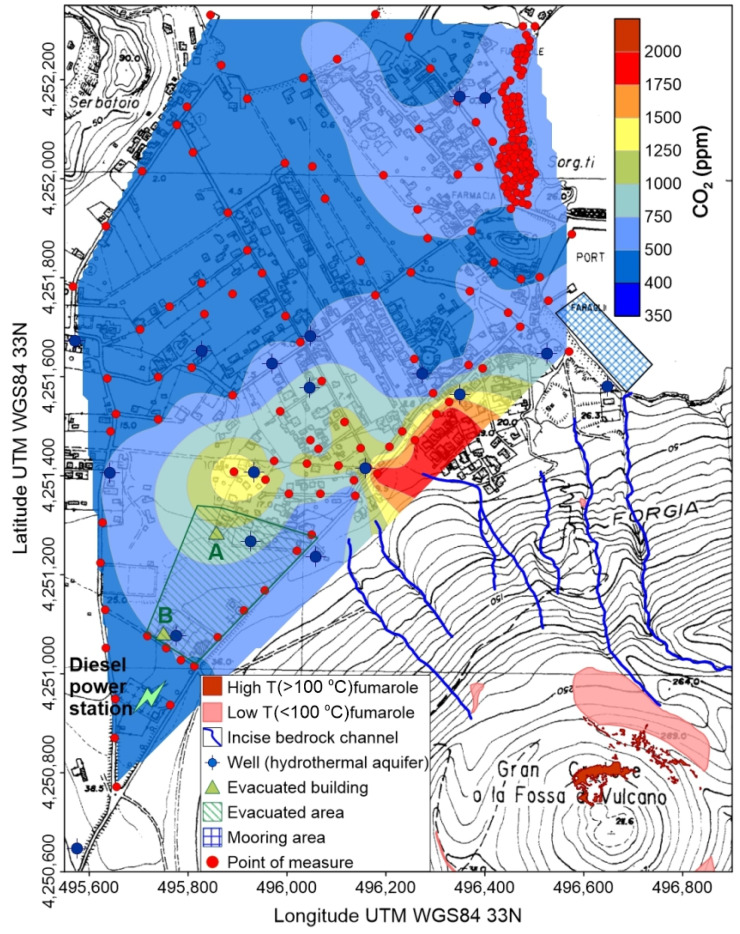
Concentration of atmospheric CO_2_ in the Vulcano Porto area on 14 November 2021. Position of fumarolic fields, incise bedrock channels, hydrothermal wells, evacuated buildings and area, summer touristic mooring area and points of measure are also shown.

Minimum, average and maximum concentrations of CO_2_ are reported in Table 2 (full data available as Supplementary Materials) and Figure 10, Figure 11, Figure 12 and Figure 13. Measures recorded at Salina and Stromboli on January 2021, and at Stromboli on April of the same year, are quite constant and show values typical of the normal CO_2_ concentration, at the net of minor variations falling within the measurement accuracy. Values exceeding the background atmospheric concentration were detected instead at Stromboli (September 2020) and Vulcano (all surveys).

At Stromboli (Figure 10) background atmospheric carbon dioxide concentrations generally exceeded 500 ppm, and two anomalous zones were characterized by values up to 700 ppm. The first one is related to a topographically depressed area immediately downhill of the cemetery, in the central portion of the village. The other one, with the highest concentrations, was located in the N corner of the inhabited area, well known for its anomalous diffuse soil CO_2_ degassing [57].

Carbon dioxide concentrations measured on January 2021 at Vulcano were generally low and within the normal atmospheric range (Figure 11), with the exception of the hydrothermally active area of East Bay (inset of Figure 11), were values up to 665 ppm were detected where high PM_2.5_ concentrations were present (Figure 5). This anomaly (peak value at 2250 ppm) was still present on October 2021 (Figure 12a), and persisted until the last survey (November 2021, Figure 12b), although differently shaped, with higher average concentrations and absence of elevated peak values. The spatial distribution of atmospheric CO_2_ concentrations on November 2021 (Figure 13), namely during the volcanic unrest started at Vulcano the previous September, shows huge variations with respect to the precedent survey (January).

Concentrations exceeding the normal atmospheric background were detected all along the south-eastern sector of the inhabited area of Vulcano Porto, bordering the NE flank of La Fossa Cone from the power station uphill, down to the port area, with maxima up to 2000 ppm.

A small hill, corresponding to the old eruptive center of the “Faraglione” [32], located immediately northward of the port, splits in two separate bodies the near ground accumulation of CO_2_, creating a secondary anomaly, with concentrations up to 750 ppm, which extends from the ”Faraglione” to the West Bay area.

## 5. Discussion

Data of PM_2.5_, magnetic susceptibility of oleander leaves and atmospheric carbon dioxide concentrations, acquired during the field surveys carried out between the second half of 2020 and 2021 at Salina, Stromboli and Vulcano islands, clearly depict the combined roles of anthropogenic and volcanic activities in driving air quality. The three surveyed areas are representative of very different environmental conditions, varying with time.

A very clean atmosphere was found at Salina island. Carbon dioxide concentration (Table 2) was equal to the 2021 world global average of 416 ppm [58], at the net of negligible fluctuations due to the instrumental precision.

Additionally, suspended particulate matter is very low all along the surveyed area (Figure 4), with average and maximum PM_2.5_ concentrations of 1.66 and 5.1 µg m^−3^, respectively (Table 2). Salina island can be then considered representative of the local background condition for atmospheric emissions, because (i). The survey was carried out in wintertime (January), when residents are generally at their minimum number, and in the specific case anthropic activities were extremely reduced due to the restrictions imposed for facing the COVID-19 pandemic; (ii) Volcanic activity ended prior to historical times, and since then vegetation has fully covered the island, stabilizing the soil and preventing the resuspension of particulate matter.

Consistently, at Salina, the magnetic susceptibility values of oleander leaves were the lowest of the whole dataset (Table 3), even diamagnetic in three sites, thus highlighting negligible concentration of magnetic particles in the samples.

On the contrary, Stromboli and Vulcano islands showed clear signs of the impact of volcanic activity, as better detailed in the following discussion.

### 5.1. Atmospheric Concentration of Particulate Matter and CO_2_ at Stromboli Island

The active volcanic vents of Stromboli continuously emit solid and gaseous particles and, when the wind blows from the north-western quadrants, which is the most common condition (Figure 2), it drives the volcanic plume over the inhabited area, where high values of both PM_2.5_ and atmospheric CO_2_ concentrations are recorded (Table 1 and Table 2 and Figure 7, Figure 8, Figure 9 and Figure 10). This condition was found during the September 2020 survey, when the background PM_2.5_ concentration was in the range 5–10 µg m^−3^, but large areas showed values between 10 and 25 µg m^−3^, exceeding the long-term WHO AQG of 5 µg m^−3^ (Figure 7). The maximum concentrations, over 50 µg m^−3^, were recorded at the southern upper limit of the village, just after a volcanic explosion (Figure 7); these values exceed the short-term WHO AQG, fixed at 20 µg m^−3^.

Different conditions were detected during the other two surveys, during which wind was blowing from the southern quadrants (Table 1), moving away the volcanic plume from the Stromboli village (Figure 8 and Figure 9). Anyway, PM_2.5_ concentrations exceeding both the long and short-term WHO AQGs were found in January 2021 (Figure 8), in the same high concentration area identified during the previous survey. Another adverse health effect of high concentrations of volcanic particulate is its accidental ingestion and consequent gastric leaching, which can produce toxic level of chemicals in organic tissues. As reported by Cangemi et al. [4], a potentially noxious As intake could derive from the accidental ingestion of about 6 g of Stromboli volcanic ashes.

From the magnetic point of view, Stromboli showed a relatively higher content of magnetic PM, especially in three sampling sites, in connection to the higher traffic intensity and the peculiar road geometry, that can prevent the dispersion of PM.

A similar behavior characterizes the spatial distribution of atmospheric CO_2_ concentration, with values exceeding the global background only found in September 2020 (Table 2 and Figure 10). It is worth noting that the maximum CO_2_ concentrations (up to 715 ppm) were found in the same area of the highest PM_2.5_ (Figure 7), suggesting that the volcanic plume, and not the soil degassing, is the source of carbon dioxide. This hypothesis is also supported by the position of the CO_2_ high concentration areas, located along the main incised bedrock channels crossing the urbanized area (Figure 10): volcanic carbon dioxide, emitted by the summit vents and heavier than the other components of the atmosphere, flows down along the bedrock channels and creates gaseous fans expanding at the base of the volcanic edifice.

### 5.2. Atmospheric Concentration of Particulate Matter and CO_2_ at Vulcano Island and Effects of the 2021 Volcanic Unrest

The PM_2.5_ survey, carried out at Vulcano on January 2021 (Figure 4), evidenced particulate matter concentrations generally comprised between the long (yearly) and short (daily) terms WHO AQG reference values, with higher concentrations in two zones: the East Bay hydrothermal area and the southern branch of the NW-SE main road crossing the inhabited area. This last anomaly still persisted in the following survey of November 2021 (Figure 3), indicating that vehicular traffic here affects at a certain extent air quality, also because many of the vehicles circulating at Vulcano are old and low-ranked in the EU classification of atmospheric emission standards for the automotive sector.

A different, geogenic source should be claimed for the very high PM_2.5_ values recorded at the East Bay on January 2021 (Figure 4), which exceeded any WHO AQG reference both for short- and long-term expositions; the last survey, on November 2021 (Figure 4), did not evidence any anomaly. Suspended atmospheric particles are here composed of micro-droplets, deriving from the condensation of the hydrothermal water vapor emitted by the diffuse soil degassing and fumaroles. In January air temperature was lower than in November (Table 1), thus more efficiently fostering the condensation of the hydrothermal vapor and, in turn, the genesis of the micro-droplets. An alternative, or co-acting cause, is the variation of the hydrothermal vapor emission rate, higher in January than in November 2021, despite the island was still experiencing a general volcanic unrest condition.

It is worth of note that the presence of very high concentrations of micro-droplets, recorded at East Bay on January 2021, represents a significant health hazard in case of epidemies caused by viruses transported by liquid atmospheric particles, as the COVID-19 pandemic. Finally, no anomalous PM_2.5_ concentrations should be referred to the emissions from the diesel power plant (Figure 4): the higher values of November were recorded in two points located on the road and upwind the plant, thus suggesting that vehicles in transit should be responsible for them.

At Vulcano island, the magnetic susceptibility of leaves was generally low (Table 3), but at the site VUL2 (Figure 8), it was slightly higher due to the presence of a thermoelectric power supply plant that clearly increased the emission and the bioaccumulation of metallic particles.

Space-time variations of atmospheric CO_2_ deserve a deep and articulate discussion, because the 2021 unrest of Vulcano island has been characterized by the higher carbon dioxide emissions here ever recorded. Prior to the onset of the unrest, on January 2021 (Figure 11), atmospheric concentrations of carbon dioxide did not exceed the world global average [58], at the net of negligible fluctuations due to the instrumental precision. The sole exception was the East Bay hydrothermal area, with a maximum value of 665 ppm (Table 2), related to the concentrated and diffuse soil degassing, but not determining any significant gas hazard condition.

On October (Figure 12a), the area with CO_2_ concentrations exceeding the normal atmospheric background was wider than in January, reaching a localized maximum of 2250 ppm in the NE corner of the rectangular area visible in the maps, delimited by a 2 m high concrete wall. Here, an excavation at its base consistently reduced the thickness of the sediment coverage of the underlying shallow (few decimeters b.g.l.) [59] thermal, CO_2_ rich aquifer [60], fostering the carbon dioxide outgassing to the atmosphere. Following the ongoing unrest, the average atmospheric CO_2_ concentration at East Bay continued to increase, reaching one month later an average value of 525 ppm, although the localized anomaly previously identified disappeared (Figure 12b).

Zooming back to a more general view on the island (Figure 13), it is evident that East Bay was the eastern sector of a wider anomalous, near-ground accumulation of CO_2_, which extends between the eruptive center of the “Faraglione”, to the south, and the West Bay, to the N. Position and shape of the anomaly suggest that it could be referred to the CO_2_ outgassed from the soil, mainly from the area closest to the “Faraglione” [32], which moved northward along the topographically depressed portion of the isthmus connecting Vulcano to Vulcanello.

The topographic drive of volcanic CO_2_, emitted by fumaroles and anomalous soil degassing, accumulating on the ground and flowing down as a pseudo-laminar flow along hillslopes, or concentrated in incised bedrock channels, is remarked by the map illustrated in Figure 13. The highest concentrations, up to 2000 ppm, are found at the base of the northern flank of La Fossa cone, where the main fumarolic field is located, and in particular at the outlet of the main stream channels. The presence of counter-slopes, acting as obstacles for the CO_2_ downflow, generated localized anomalies, as the secondary concentration peak located westward of the main one. It is worth of note that a mooring area, active during the touristic season (blue gridded polygon in Figure 13), is located at the outlet of two incised bedrock channels, whose drainage area comprehends part of the main fumarolic field, thus determining potential gas hazard conditions.

This mechanism was responsible of the evacuation of building A (Figure 13) in the morning of 12 October 2021: here, due to a retaining wall 3 m high, located immediately downslope the building, CO_2_ concentrations up to 100% were recorded at ground level. In the late evening of the same day, another building (B in Figure 13), after measuring indoor concentrations up to 5000 ppm, was evacuated as well as, in the following days, a larger area (green lined polygon in Figure 13) comprehending the two buildings.

**Figure 14:**
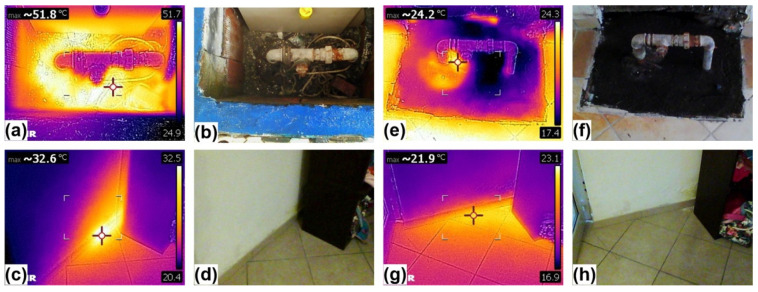
Visible and IR images of the well head and of the adjacent wall (from the interior) of building B (Figure 10). (**a**) IR image of well head taken on 12 October 2021; (**b**) Corresponding visible image; (**c**) IR image of the wall taken on 12 October 2021; (**d**) Corresponding visible image; (**e**) IR image of well head taken on 13 November 2021; (**f**) Corresponding visible image; (**g**) IR image of the wall taken on 13 November 2021; (**h**) Corresponding visible image.

The high CO_2_ concentration recorded in building B cannot be explained by the topographic drive, because it is located on an open decline without obstructions: here, carbon dioxide was vented in huge amounts from a water well, drilled strictly close to the main entrance of the building (Figure 14), together with water vapor that heated the drill head and the adjacent wall (Figure 14a–d). A strong sound of boiling water was clearly audible (water table at 35 m b.g.l.) but, surprisingly, the temperature of water pumped from the well was 66 °C, definitely below the boiling point of water, thus suggesting that the sound was generated by an intense CO_2_ bubbling.

**Figure 15:**
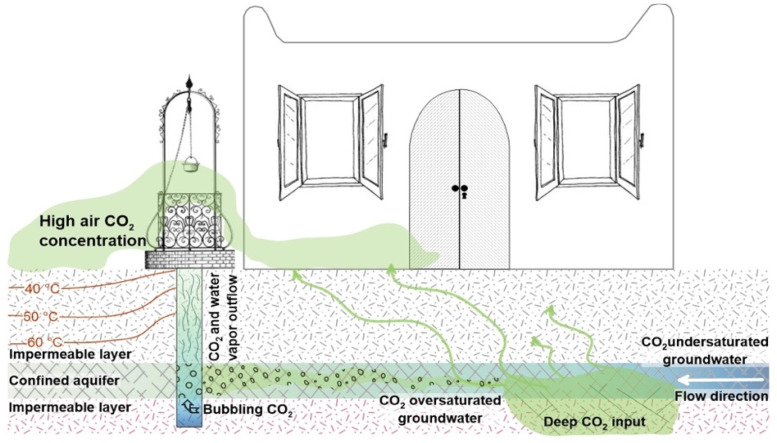
Conceptual scheme of the atmospheric CO_2_ and water vapor degassing from the Vulcano shallow, thermal aquifer.

Following this observation, we propose that the aquifer was flashed by volcanic CO_2_, carrying water vapor to the surface (Figure 15).

**Figure 16:**
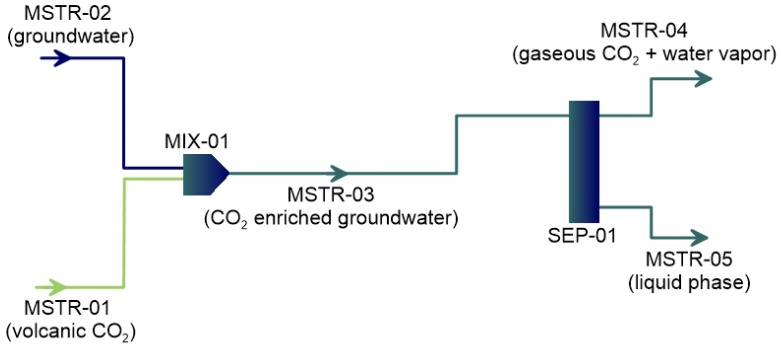
Flowsheet used in the DWSIM computer program for modeling the CO_2_ flashing of the Vulcano aquifer.

The CO_2_ flashing has been reproduced by a process simulation, by means of tools commonly used in petroleum & petrochemical engineering (DWSIM 6.7.1, see Methods), according to the flowsheet reported in Figure 16. The flashing model consists of two input streams, simulating the groundwater flow (MSTR-02) intercepted by the deep CO_2_ degassing (MSTR-01), mixing together (MIX-01) in a stream (MSTR-03), which is the input of an adiabatic flash separator (SEP-01), where the mixed stream is separated in gas (MSTR-04) and liquid (MSTR-05) phases, at given input temperature and pressure. The gas-liquid equilibrium has been calculated according to the Peng-Robinson state equation, implemented in the DWSIM code library, which well fits the CO_2_ absorption in water.

**Figure 17:**
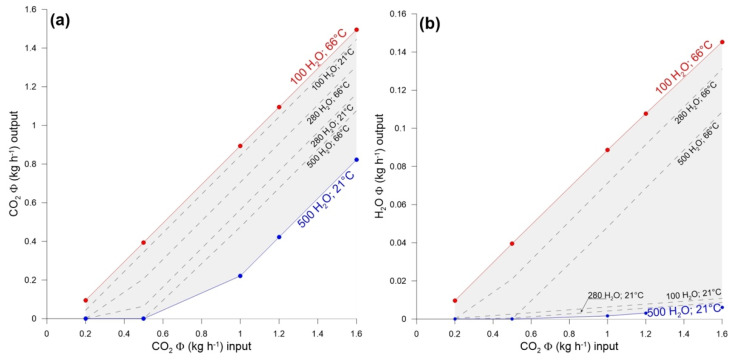
(**a**) Simulation of CO_2_ flux from the Vucano shallow thermal aquifer, flashed with increasing inputs of volcanic carbon dioxide, at 21 and 66 °C, and for groundwater flow rates from 100 to 500 kg h^−1^. (**b**) Simulation of water vapor flux from the Vucano shallow thermal aquifer, flashed with increasing inputs of volcanic carbon dioxide, at 21 and 66 °C, and for groundwater flow rates from 100 to 500 kg h^−1^. All flux values are referred to a unit surface of 1 m^2^.

We performed different simulations, considering different water temperatures and flows, according to literature data [59], and CO_2_ fluxes, up to a maximum of 1.6 kg h^−1^, compatible with the higher values of soil degassing recorded during the volcanic crisis [61]: calculations are referred to unit surface areas of 1 m^2^. Results are presented in the graphs of Figure 17, where in (a) and (b) are plotted the fluxes of CO_2_ and water vapor outgassed from groundwater versus different inputs of volcanic CO_2_, respectively.

As evidenced in (a), at groundwater temperatures as high as those found in the B building well (66 °C), from 40% to 80% (according to the groundwater flux) of the volcanic CO_2_ passes through the aquifer and is released to the atmosphere, coupled to a water vapor flux one order of magnitude less (Figure 17b). At normal environmental temperature (21 °C), the CO_2_ outflow is more sensitive to the groundwater flow, being CO_2_ completely dissolved in the liquid phase for volcanic inputs lower than 0.5 kg h^−1^ and groundwater flow rates of 500 kg h^−1^. As expected, the water vapor tension is very low at ambient temperature (Figure 17b), and no stripping effects due to carbon dioxide inlets are noticed, even at very high CO_2_ inlets.

Our flashing model supports the hypothesis that CO_2_ outgassed from the aquifer significantly contribute to create the dangerous ground-level accumulations previously discussed, especially in the close proximity of water wells, carrying up to its 8%_w_ of water vapor, responsible for the thermal anomalies detected around the wells (Figure 14a–c). Two more observations support this interpretation: (i) Another survey, carried out one month later (13 November 2021) on B building, after the owner had roughly sealed the well introducing textile material inside, revealed a drastic reduction of the well head and adjacent wall temperatures (Figure 14e–h), and of the outdoor/indoor atmospheric CO_2_ concentrations (< 700 ppm); at the same time at building A, without interventions, no significant concentrations changes were detected.; (ii) As displayed in Figure 13 the spatial distribution of anomalous CO_2_ concentrations well fits that one of the wells extracting groundwater with a marked hydrothermal character [59].

A final, important consideration concerns timing and duration of atmospheric CO_2_ anomalies. As described in the introduction, the onset of serious gas hazard conditions in the Vulcano Porto area dates at 12 October 2021, when the first two buildings were evacuated. The day after high CO_2_ concentrations were detected in a restricted area of East Bay but, one month later, in spite of the perduring unrest conditions and a general increment of the background values, this high intensity anomaly disappeared (Figure 12). These facts suggest that impulsive, localized and short-lasting events modulate a diffuse, long-lasting CO_2_ degassing, which has significantly incremented during the volcanic unrest.

**Figure 18:**
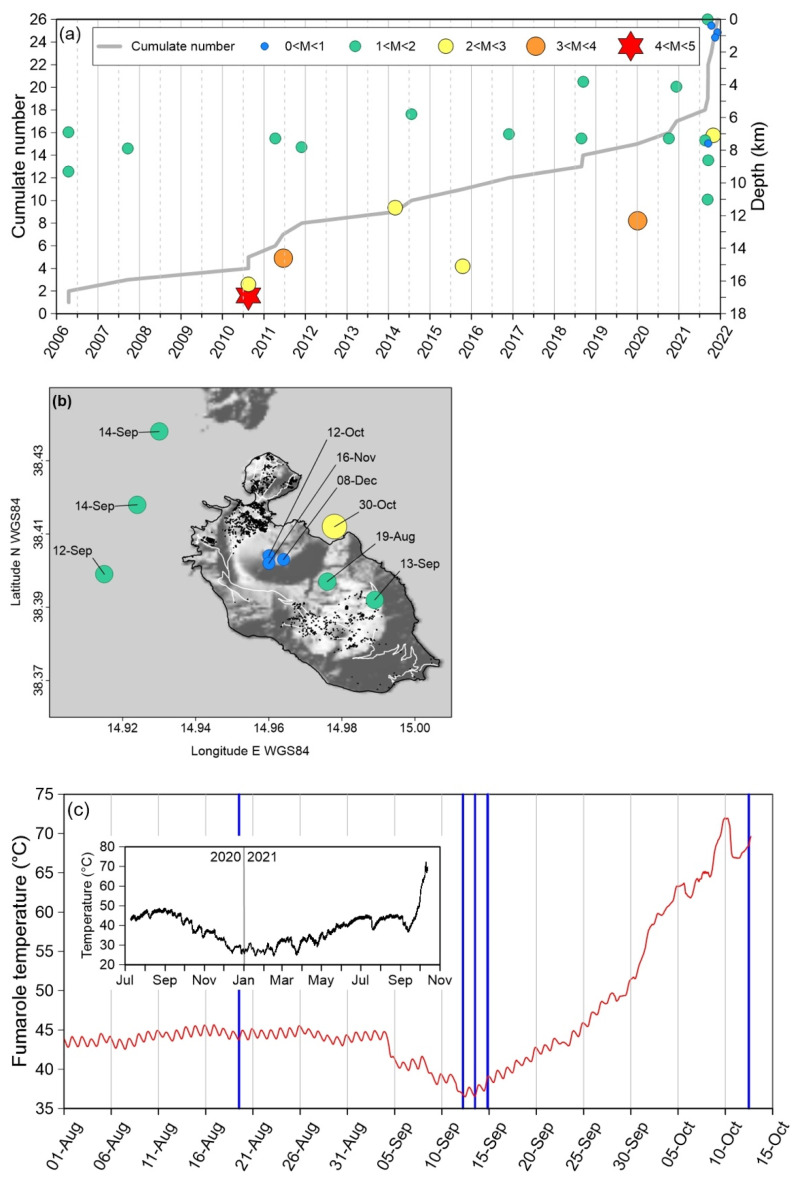
(**a**) Cumulate number, depth and magnitude of the earthquakes occurred at Vulcano island, and its immediate surroundings, in the period 2006–2021 [63,64]. (**b**) Epicentral location, date and magnitude (ML, between brackets) of the earthquakes occurred between August and November 2021 at Vulcano island and its immediate surroundings. The background DEM is from [65], earthquakes localization from [63,64]. (**c**) Soil temperature (red line), measured at 50 cm depth between August and October 2021, of a fumarole located on the NE rim of la Fossa cone, with indication of the earthquakes occurred in the same period (blue vertical bars); in the inset, fumarole temperature in the period July 2020–October 2021.

Previous studies demonstrated that the magmatic-hydrothermal system of Vulcano island is sensitive to tectonic stress and strain, to which reacts with degassing pulses as more relevant as higher is its volcanic activity state [62]. The 2021 unrest is a paradigmatic example of such behavior. Figure 18a illustrates the time distribution of seismicity, in the immediate surroundings of Vulcano, during the last 15 years. The most energetic earthquake of the period occurred on August 2010, causing some degassing anomalies, without perturbing the general state of the volcano [62]. After that episode, tectonic seismicity went on regularly until the second half of 2021, when a sequence of 9 earthquakes, most of which with shallow hypocenters, hit the island (Figure 18b).

Four of them occurred between 12 and 13 September, shortly after followed by a huge increment in the release of volcanic gases. One evidence is in a steep (more than 30 °C in less than one month) increase of fumarole temperatures, as recorded by a monitoring station located on the NE rim of la Fossa cone (Figure 18c).

Degassing further increased since the end of September, as testified by the steeper slope of the fumarole temperature curve and the disappearing of the circadian thermal cycle (Figure 18c), indicating that the endogenous, vapor-driven thermal flux was obliterating the effects of the thermal exchange with the atmosphere. This process culminated on 10 October, when the thermal signal started to experience a short-term variability, suggesting that a fracturing process was perturbing the degassing regime. On 12 October at 11:40 local time, a very shallow earthquake (0.4 km b.s.l. [63]) occurred on the W flank of la Fossa cone (Figure 18b). Few hours later anomalous outdoor and indoor CO_2_ concentrations were detected in this area, leading to the evacuation of buildings A and B (see previous discussion), and the day after the survey at East Bay individuated a high intensity CO_2_ anomaly, later disappeared (Figure 12a,b).

These observations indicates that crustal stress variations and seismic strain releases deeply interact with shallow volcanic degassing systems, leading to potentially lethal gas hazard conditions, especially shortly after very shallow earthquakes.

### 5.3. Magnetic Properties of Oleander Leaves

Muhammad et al. [66], showed that it is inappropriate to compare the concentration-dependent magnetic parameters of leaves of different plant species, and that several factors influence their values, beyond the distance from the emission sources. Thus, the results are here compared with those already available for oleander leaves. In Moreno et al. [67] oleander leaves showed mean value χ = 18.34 × 10^−8^ m^3^/kg and were considered efficient bioaccumulators, second only to *Quercus ilex*, as appreciated measuring and comparing leaves of various tree and shrubs species exposed side by side in trafficked roads of Rome. In Winkler et al., 2022, oleander leaves were sampled in the Gardens of a historical Villa, were they showed χ = 2.2 and 1.1 × 10^−8^ m^3^/kg in September and December, respectively, being among the lowest values reported for the whole dataset, as mainly ascribed to influence of the distance from the road. So, it is possible to argue that the values measured in this study are generally low, and reflect a minor bioaccumulation of magnetic particles even in the sites characterized by the higher values of χ, which were observed in proximity of the Scari pier in Stromboli and near the thermoelectric power plant in Vulcano.

It was possible to define reliable hysteresis parameters only for the few samples with χ > 1 × 10^−8^ m^3^/kg. Conversely to the previous studies that were carried out in urban traffic contexts, the “Day plot” indicated no influence from vehicular brakes, and the oleander points fell in the central-upper zones of the plot, indicating that anthropic exhausts and natural emission sources determine the magnetic fraction of PM in Aeolian islands.

It is worthwhile to note that at Stromboli (Figure 8), the lowest values were observed in the highest part of the village, farer from the most trafficked road but closer to the source of volcanic particulate (the summit vents), indicating that the natural background is much lower than the anthropogenic component.

In summary, the magnetic properties of the oleander leaves sampled in Salina, Stromboli and Vulcano islands pinpointed low concentrations of magnetic particles even in the sites where the concentration dependent magnetic particles were highest, in presence of exhaust fuel emissions from vehicles and thermoelectric power plants.

## 6. Conclusions

Our study presents the first data on atmospheric concentrations of PM_2.5_ and CO_2_ at Salina, Stromboli and Vulcano islands, in the Aeolian Archipelago (South Mediterranean), integrating direct spot measures and analyses of the magnetic properties of oleander leaves, where atmospheric particulate matter bioaccumulates. These investigations are very important for opposite reasons. On one hand, the archipelago belongs to the UNESCO World Heritage, and preserving high standards of environmental quality is fundamental for keeping this status. Conversely, natural phenomena as volcanic activity pose serious threats to human health, and high atmospheric concentrations of volcanic particulate and carbon dioxide are among the most relevant of these.

Our results highlighted that no significant anthropogenic sources for atmospheric particulate are active in the investigated islands, at the net of a scarcely relevant contribution due to the combustion of gasoline for automotive purposes or to metallic emissions related to a thermoelectric power station. Under the perspective of a continuous enhancement of the environmental quality of these sites, the complete conversion of the circulating automotive pool to electrically powered units seems then desirable. Conversely, volcanic activity determines potentially noxious concentrations of suspended fine ashes at Stromboli, and of micro water droplets in the hydrothermally active area of East Bay at Vulcano. These preliminary results indicate that attention on the evolution of PM_2.5_ concentrations during the recurring increments of volcanic activity is needed.

Similar considerations originate from the interpretation of atmospheric CO_2_ concentration data. Carbon dioxide, emitted by the summit active volcanic vents (Stromboli) and from the main fumarolic field (Vulcano), flows along the incised bedrock channel network and later expand at their outlets in the coastal area, forming local hazardous accumulations fostered by favorable topographic conditions (counter-slopes). The same gas hazard condition can be generated by the intense diffuse CO_2_ soil degassing, as in the East Bay area at Vulcano. A third, unconventional source identified by our study is the direct CO_2_ degassing from the shallow hydrothermal aquifer at Vulcano, possibly triggered by shallow local earthquakes, which can lead to dangerous atmospheric concentrations in proximity of drilled or hand-carved water wells.

These results are of general interest for the adoption of gas hazard mitigation actions in active volcanic areas where inhabited areas are located in close proximity of, or partially or totally coincide with, concentrated or diffused CO_2_ degassing sources.

## Figures and Tables

**Table 1:** Date and environmental conditions during surveys; N, T and Rh number of measurements, air temperature and relative humidity, respectively, P atmospheric pressure, Wdir and Ws wind direction and speed, respectively. The number of explosions per hour (Expl) [41] is also given (limited to Stromboli, n.a. is not applicable). All values are daily averages.

Site (Date)	N	T (°C)	Rh (%)	P (hPa)	Wdir (°)	Ws (m s^−1^)	Expl
Salina (4 January 2021)	80	9.4	61	1010	SSW	3.3	n.a.
Stromboli (23 September 2020)	93	26.6	74	1011	WNW	2.0	10–15
Stromboli (5 January 2021)	78	13.6	60	1012	SSW	2.5	10–15
Stromboli (2 April 2021)	168	15.4	83	1015	SW	4.6	5–9
Vulcano (6 April 2021)	93	15.5	62	1013	S	2.3	n.a.
Vulcano (13 October 2021)	115	18.5	79	1009	WNW	1.0	n.a.
Vulcano (13 November 2021)	189	18.5	86	1015	S	3.0	n.a.

**Table 2:** Minimum, average, maximum and standard deviation of PM_2.5_ and CO_2_ measured during the different surveys at Salina, Stromboli and Vulcano islands (n.a. is not applicable).

Site (Date)	PM_2.5_ (µg m^−3^)				CO_2_ (ppm)			
	Min	Mean	Max	σ	Min	Mean	Max	σ
SAL (4 January 2021)	0.3	1.66	5.1	1.02	400	402	433	7.3
STR (23 September 2020)	4.7	12.8	135	14.1	461	534	715	52.7
STR (5 January 2021)	0.6	2.93	44.7	5.16	400	402	429	5.74
STR (2 April 2021)	4.1	7.81	14.5	1.64	constant at 410 ppm			
VUL (6 January 2021)	2.3	13.6	656	68.6	400	408	665	37.5
VUL (13 October 2021)	n.a.	n.a.	n.a.	n.a.	444	522	2250	170
VUL (13 November 2021)	3.6	6.39	29.5	2.58	434	587	2044	246

**Table 3:** Number of samples (N), minimum (MIN), maximum (MAX) and average (AVE) magnetic susceptibility values (χ, 10^−8^ m^3^ kg^−1^) of the oleander leaves’ samples collected at Salina, Stromboli and Vulcano.

Site	N	MIN	MAX	AVE χ
Salina	8	−0.23	0.49	0.16
Stromboli	5	0.34	2.24	1.22
Vulcano	4	−0.05	2.51	0.71

**Table 4:** Sample Id, hysteresis parameters and their ratios for the oleander leaves sampled and collected at Stromboli and Vulcano islands. The samples were selected according to their magnetic susceptibility values.

Id	Ms (mAm^2^/kg)	Mrs (mAm^2^/kg)	Bc (mT)	Bcr (mT)	Bcr/Bc	Mrs/Ms
STR2	1.32	0.38	23.0	47.6	2.07	0.29
STR4	0.63	0.12	23.0	43.8	1.91	0.19
STR5	1.44	0.33	17.6	38.5	2.18	0.23
VUL2	1.51	0.33	14.7	43.1	2.92	0.22

## Data Availability

All data related to this study are available online as Supplementary Materials.

## Supplementary Materials

The following supporting information can be downloaded at: https://www.mdpi.com/article/10.3390/ijerph19084833/s1.
